# Toward Next‐Generation Teratology: Understanding Developmental Vulnerability Through Evolutionary and Comparative Genomics

**DOI:** 10.1002/cga.70074

**Published:** 2026-08-26

**Authors:** Tetsuo Kon

**Affiliations:** ^1^ Department of Neurosciences and Developmental Biology University of Vienna Vienna Austria

**Keywords:** developmental constraints, environmental mismatch, evolutionary and comparative genomics, evolutionary trade‐offs, next‐generation teratology

## Abstract

Teratology, the study of congenital anomalies, has developed from a descriptive discipline into a mechanistic science focused on the proximate causes of developmental defects, meaning *how* such anomalies arise during development. However, a comprehensive understanding requires not only this mechanistic insight but also an evolutionary perspective on *why* developmental systems are susceptible to disruption, often following recurring yet diverse patterns. To better grasp this susceptibility, it is important to consider evolutionary contexts, including concepts such as evolutionary trade‐offs, environmental mismatch, and the intrinsic properties of developmental constraints. This perspective is now greatly enriched by advances in genome sequencing technologies. In this review, we revisit the concept of teratology within an evolutionary framework, and highlight the profound impacts of modern genome sequencing technologies, which are reshaping our view of teratology, evolutionary biology, and life science as a whole. We proceed to explore developments in three key research areas, each corresponding to a different evolutionary timescale: vertebrate appendage patterning and its evolutionary origins, whole‐genome duplication and ohnolog evolution as sources of developmental complexity and dosage sensitivity, and deeply conserved chromosomal organization and transposable‐element dynamics across animal evolution. Finally, we propose a future direction for “*next‐generation teratology*” that fully integrates evolutionary and comparative genomics with conventional teratology to provide novel approaches to understand, diagnose, and ultimately prevent congenital anomalies.

## 1 Introduction

Teratology is classically defined as the study of congenital anomalies (birth defects) which are structural or functional abnormalities present at birth [1]. Congenital anomalies arise from various genetic, biological, and environmental factors. Congenital anomalies represent a significant public health concern because approximately 2%–3% of newborns present detectable anatomical abnormalities [2, 3, 4, 5, 6, 7]. Understanding the mechanisms of these congenital anomalies is therefore crucial for their prevention, diagnosis, and management.

Classic teratology has focused on the detailed description of congenital anomalies and the identification of environmental teratogens, such as drugs or infections that disrupt normal human embryonic development. However, it has long been recognized that congenital anomalies also arise from genetic changes, and increasingly, the distinction between environmental and genetic causes has blurred [8, 9]. Elucidating the genetic basis of congenital anomalies is thus essential for improving molecular diagnosis, risk assessment, genetic counseling, and clinical management.

Clinical genomic testing has already transformed the diagnosis of congenital anomalies. Karyotyping and chromosomal microarray analysis identify aneuploidies and pathogenic copy‐number variants, whereas exome sequencing has substantially improved the detection of disease‐causing coding variants, particularly in patients or fetuses with multiple structural anomalies [10]. Genome sequencing further offers the potential to detect a broader spectrum of variation, including structural rearrangements and variants outside protein‐coding regions. Nevertheless, a considerable proportion of congenital anomalies remain unexplained because current approaches incompletely capture complex structural variants, mosaicism, repetitive genomic regions, noncoding regulatory variants, and gene–environment interactions [11]. Moreover, the interpretation of variants of uncertain significance remains a major clinical challenge. Comparative and evolutionary genomics can complement clinical sequencing by identifying deeply conserved genes, regulatory elements, and chromosomal architectures in which genomic alterations are more likely to have developmental consequences.

One particularly clear example comes from ciliopathy research. Comparative genomic analyses of ciliated or flagellated and nonciliated eukaryotes identified a conserved candidate flagellar and basal‐body proteome and, through subsequent mutation analysis, led to the identification of BBS5 as a Bardet‐Biedl syndrome gene [12]. A related strategy combining comparative genomics, gene‐expression analysis, and homozygosity mapping subsequently identified BBS9 [13], while MKS1, encoding a component of the conserved basal‐body proteome, was shown to be mutated in Meckel syndrome [14]. Together, these studies linked evolutionarily conserved ciliary components to human developmental disorders characterized by polydactyly, renal malformations or dysplasia, and other congenital abnormalities. Comparative genomics therefore contributed not only to understanding ciliary biology but also directly to disease‐gene discovery in ciliopathies and related congenital anomaly syndromes.

Despite these successes in elucidating proximate causes of specific congenital anomalies, a different level of explanation is needed: *Why* are developmental systems so vulnerable to disruption? Evolutionary theory provides a crucial framework for addressing this question [15, 16, 17, 18, 19, 20]. To address this “*why*,” we need to look beyond proximate causes and consider how developmental systems have been shaped by evolution [16, 17]. Genomes are not static blueprints but are evolving ones that change with each generation, producing a spectrum of phenotypes [19, 21, 22]. Although dramatic phenotypic changes are often deleterious or rarely beneficial, a substantial portion of phenotypic variation observed in natural populations may have little or no impact on fitness and thus can be nearly neutral in evolutionary terms [23, 24]. This view suggests that the broad spectrum of congenital anomalies observed in clinical settings is not simply the result of developmental errors, but may also reflect part of the natural variation on which evolutionary change can act.

The importance of studying evolutionary processes using high‐quality genome assemblies has long been recognized. However, it is only recently that advances in sequencing and assembly technologies have made such genome‐scale evolutionary analysis practical [25]. Over the past few decades, remarkable progress in both experimental and computational methods has enabled the rapid generation of chromosome‐level genome assemblies for a wide range of animal species. Large‐scale comparison of these genome sequences (“comparative genomics”) is beginning to uncover the molecular and evolutionary bases of distinctive animal traits [26, 27, 28, 29, 30, 31, 32, 33, 34, 35] (Table 1), as well as the principles of chromosome evolution that are conserved across evolutionarily distant animals [49, 50, 51, 52]. Traditionally, life science research has centered on a few model organisms, but recent advances in genome and gene editing technologies now allow the study of diverse species‐specific traits.

**TABLE 1:** Representative animal phenotypes and shared genes implicated in human congenital anomalies or diseases.

Animal	Phenotype	Gene	Human disorder associated with the same gene^a^	OMIM ID
White tiger	White coat color [26]	SLC45A2	Albinism, oculocutaneous, type IV [36]	606 574
Belgian blue cattle	Double‐muscling [27]	MSTN	Muscle hypertrophy [37]	614 160
Dog	Long hair [28]	FGF5	Trichomegaly [38]	190 330
Bat	Wing formation [29]	PRX1	Agnathia‐otocephaly complex [39, 40]	202 650
Darwin's finches	Beak shape diversity [30]	ALX1	Frontonasal dysplasia 3 [41, 42]	613 456
Snake	Infrared detection [31]	TRPA1	Episodic pain syndrome, familial, 1 [43]	615 040
Snake	Limb loss [32]	SHH	Limb malformations [44, 45]	NA
Goldfish	Telescope eye [33]	LRP2	Donnai–Barrow syndrome [46]	222 448
African cichlid	Jaw shape diversity [34]	BMP4	Orofacial cleft 11 [47]	600 625
Stickleback	Pelvic reduction [35]	PITX1	Clubfoot, congenital, with or without deficiency of long bones and/or mirror‐image polydactyly [48]	119 800

^a^ The animal phenotypes listed in this table are evolved, naturally occurring, or domestication‐associated traits and should not be regarded as direct equivalents of human congenital anomalies or diseases. The indicated relationships are based on shared genes or developmental pathways and are intended to illustrate common developmental and genomic principles.

**FIGURE 1:**
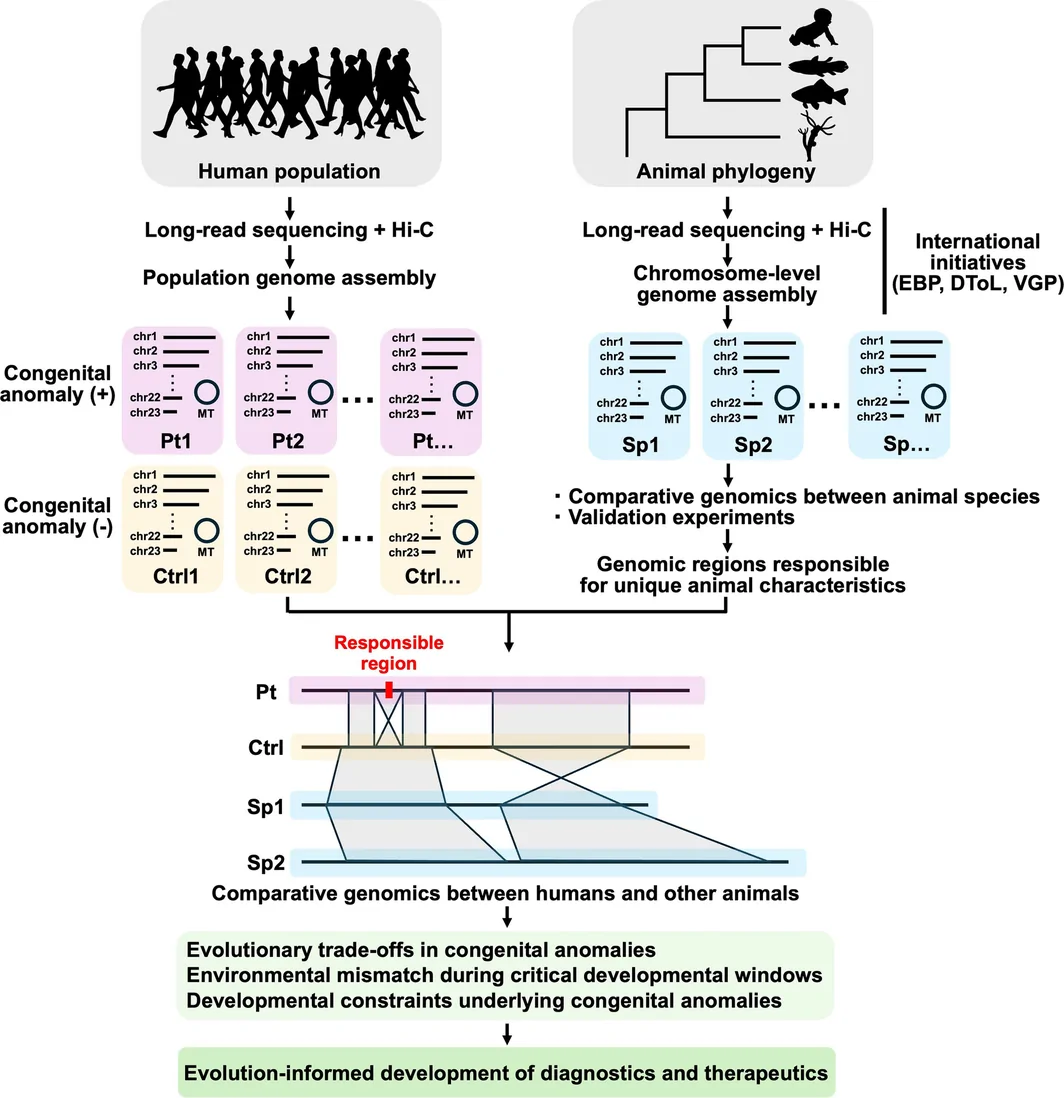
Next‐generation teratology based on evolutionary and comparative genomics. The increasing availability of chromosome‐level genome assemblies has opened new possibilities for population genome assembly approaches that determine individual genome structures in an unbiased manner, without relying on reference genome guidance [53, 54]. Meanwhile, international initiatives in evolutionary and comparative genomics have driven the generation of chromosome‐scale assemblies across diverse animal clades [55, 56, 57]. These resources allow for high‐resolution, quantitative analyses of congenital anomalies in an evolutionary context. The integration of these insights can lead to the discovery of the roles of evolutionary trade‐offs, environmental mismatch, and developmental constraints in shaping congenital anomalies. Ultimately, this approach will guide the evolution‐informed development of diagnostics and therapeutics. Ctrl, control (unaffected individual); DToL, Darwin Tree of Life Project; EBP, Earth BioGenome Project; Pt, patient (individual with a congenital anomaly); Sp, species; VGP, Vertebrate Genomes Project. Animal silhouettes are from PhyloPic [58].

Here, we use the term “next‐generation teratology” to denote an integrative framework that combines conventional mechanistic teratology and clinical genomics with evolutionary theory and comparative genomics (Figure 1). Its central aim is not only to identify the immediate genetic or environmental causes of congenital anomalies, but also to explain why particular developmental systems, genomic regions, and phenotypic outcomes are repeatedly vulnerable to perturbation. This framework therefore connects clinical observations, gene–environment interactions, developmental mechanisms, and cross‐species genomic comparisons. In this review, we revisit the concept of teratology within an evolutionary framework. We then explore key examples from recent research that offer novel perspectives for the field. Finally, we propose a forward‐looking perspective on “next‐generation teratology,” built on the full integration of an evolutionary and comparative genomics perspective.

## 2 Evolutionary Framework for Teratology

To understand *why* developmental systems are inherently susceptible to disruption, it is necessary to move beyond proximate causes and adopt an evolutionary perspective. Within this framework, congenital anomalies can be examined in relation to a broader spectrum of phenotypic variation shaped and constrained by deep evolutionary history. However, normal variation, minor anomalies, and clinically significant congenital anomalies are distinct categories and should not be regarded as equivalent. Moreover, disruptions of embryonic development do not necessarily result in anomalies observed at birth. Depending on their timing and severity, they may instead lead to embryonic or fetal loss. The evolutionary examples discussed here are therefore intended to illuminate shared developmental mechanisms and constraints, rather than to equate normal variation with clinically significant disease. To consider how an evolutionary framework may extend current approaches in teratology, it is worthwhile to revisit the historical development of this field.

### 2.1 From Description to Mechanism: A Historical View of Teratology

The history of teratology reflects a progressive shift from descriptive observation to mechanistic investigation of congenital anomalies [1, 59]. Although sporadic records of congenital anomalies date back to before the common era [60], scientific approaches emerged in the 18th and 19th centuries, driven by pioneers such as Étienne Geoffroy Saint‐Hilaire, who performed some of the first experimental analyses by manipulating chick embryos, and his son Isidore, who introduced the term “teratology.” Saint‐Hilaire and early naturalists studied congenital anomalies to deduce the normal laws of organismal construction, implicitly touching upon concepts of developmental rules and constraints [1, 59, 61]. Historical efforts to systematically document human development and congenital anomalies are reflected in prominent collections around the world such as the pathological‐anatomical collection at the *Narrenturm* in Vienna, Austria (Figure S1A), which was significantly expanded in the 19th century under the direction of Carl von Rokitansky [62, 63, 64, 65] (Figure S1B). Rokitansky is often referred to as the Linné of pathological anatomy for his efforts to establish a systematic classification of human pathological diseases, including congenital anomalies [66]. Rokitansky's name is also associated with Mayer–Rokitansky–Küster–Hauser syndrome, a congenital Müllerian anomaly characterized by agenesis or aplasia of the uterus and upper vagina [67].

Modern experimental teratology began in the 20th century. Fred Hale found that vitamin A deficiency in pregnant pigs causes specific birth defects such as anophthalmia, as well as cleft lip and palate [68]. This showed that environmental factors can consistently interfere with normal development. Josef Warkany later demonstrated that various chemicals and nutritional deficiencies could cause birth defects in mammals through controlled experiments [69, 70, 71]. Because of his pioneering work linking specific teratogens to developmental outcomes, he is widely regarded as the father of experimental teratology [59, 72, 73]. In the period from the late 1950s to the early 1960s, the thalidomide disaster brought the field to public attention, because the drug caused severe congenital limb malformations (phocomelia) in more than 10 000 children worldwide, leading to the development of modern drug safety regulations [74]. This period resulted in James G. Wilson's “Six Principles of Teratology,” which continue to guide research in the field today [75]. These principles emphasize genotype–environment interactions, developmental stage‐specific susceptibility, mechanisms of teratogenic action, the access of adverse influences to developing tissues, the major manifestations of abnormal development, and dose–response relationships [1, 75].

The thalidomide disaster also illustrates an important limitation in translating teratological findings across species. Standard mouse and rat models are relatively resistant to thalidomide‐induced limb malformations, whereas rabbits and nonhuman primates show greater susceptibility and phenotypes more similar to those observed in humans. These differences may reflect variation in drug metabolism, developmental timing, molecular targets, and embryonic responses among species [74]. Consequently, the absence of an adverse phenotype in a single laboratory species does not necessarily indicate safety in humans, underscoring the importance of using multiple experimental models when assessing teratogenic risk.

During the latter half of the 20th century, significant progress was made in understanding the genetic basis of congenital anomalies [76, 77]. The development of Sanger sequencing in 1977 provided a means to determine DNA sequences at nucleotide resolution [78]. In the following decades, the expansion of genetic markers, linkage analysis, and positional cloning approaches enabled researchers to systematically map, isolate, and sequence disease‐causing genes. Major breakthroughs during this time, such as identifying mutations in fibroblast growth factor receptor 3 (FGFR3) for achondroplasia [79] and in treacle ribosome biogenesis factor 1 (TCOF1) for Treacher Collins syndrome [80], firmly established that single‐gene defects could underlie specific congenital anomalies. Together, these advances reinforced the genetic foundation of many developmental disorders and led to the inclusion of medical genetics in the study of congenital anomalies.

While recent advances in teratology have greatly clarified the proximate causes [81, 82, 83] that explain the immediate “*how*” of congenital anomalies like genetic mutations or environmental exposures, they have often overlooked a different level of explanation: ultimate causes [81, 82, 83], which ask “*why*” developmental systems are inherently vulnerable and fail in such predictable ways.

### 2.2 Beyond Proximate Causes: Answering “Why” With Evolutionary Theory

As noted above, teratology has advanced significantly by elucidating the proximate causes of congenital anomalies, specifically, *how* developmental abnormalities occur. However, to answer the ultimate question of *why* developmental systems are so susceptible to disruption, it is essential to incorporate an evolutionary perspective. From this viewpoint, organisms should not be understood as perfectly designed machines, but rather as complex outcomes of evolutionary compromises that tend to favor reproductive success over health or robustness [16, 17, 19]. Therefore, vulnerabilities in the developmental process may arise, at least in part, from these evolutionary compromises.

Several key evolutionary concepts help explain this vulnerability. One important concept is the evolutionary trade‐offs. For instance, through antagonistic pleiotropy, in which a single gene affects multiple traits, a gene that has a beneficial effect in one aspect may increase the risk of abnormalities in another [19, 84, 85]. Another relevant concept is the evolutionary mismatch with modern environments. Human developmental and physiological systems evolved under ancestral environments and are not always robust to evolutionarily novel factors unique to modern society, such as certain chemicals or nutritional conditions [86, 87].

Congenital anomalies often appear in specific and predictable patterns, and this can be explained by two key features of the developmental process: developmental constraints [88] and canalization. Developmental constraints arise from the intrinsic structure and logic of the developmental system itself. These constraints limit the range of possible phenotypic variations, which helps explain why congenital anomalies are not random but tend to follow certain patterns [88]. Canalization, a concept introduced by Waddington [89], refers to the ability of a developmental pathway to produce a consistent outcome despite genetic or environmental disturbances. When this buffering system is overwhelmed, for example by a mutation or a strong environmental factor, development can be pushed off its usual track. This is known as decanalization [90], and it can lead to a congenital anomaly. This perspective aligns with modern findings in teratology, which reveal that genetic and environmental influences on congenital anomalies are closely interconnected [1].

Thus, by integrating evolutionary concepts such as evolutionary trade‐offs, environmental mismatch, and the intrinsic properties of developmental constraints and canalization, we can understand developmental vulnerability not simply as an error, but as a recurring outcome shaped through deep evolutionary history. This focus on ultimate causes offers a deeper understanding of the origins of congenital anomalies.

In this context, genome sequencing technologies provide a practical foundation for applying evolutionary and comparative genomics to teratology. In particular, long‐read sequencing, Hi‐C scaffolding, and haplotype‐resolved genome assembly (Figure S2) now make it possible to analyze structural variants, repetitive regions, noncoding regulatory elements, and chromosome‐scale genome organization more comprehensively. These advances are relevant to congenital anomaly research because many unexplained cases may involve genomic changes that are difficult to detect or interpret using conventional approaches. The increasing availability of chromosome‐level assemblies from both humans and diverse animal species now provides a foundation for comparing patient genome structures with evolutionarily conserved genomic architectures (Figure 1 and Supporting Information Note).

## 3 Evolutionary and Comparative Genomics Approaches to Developmental Systems

**FIGURE 2:**
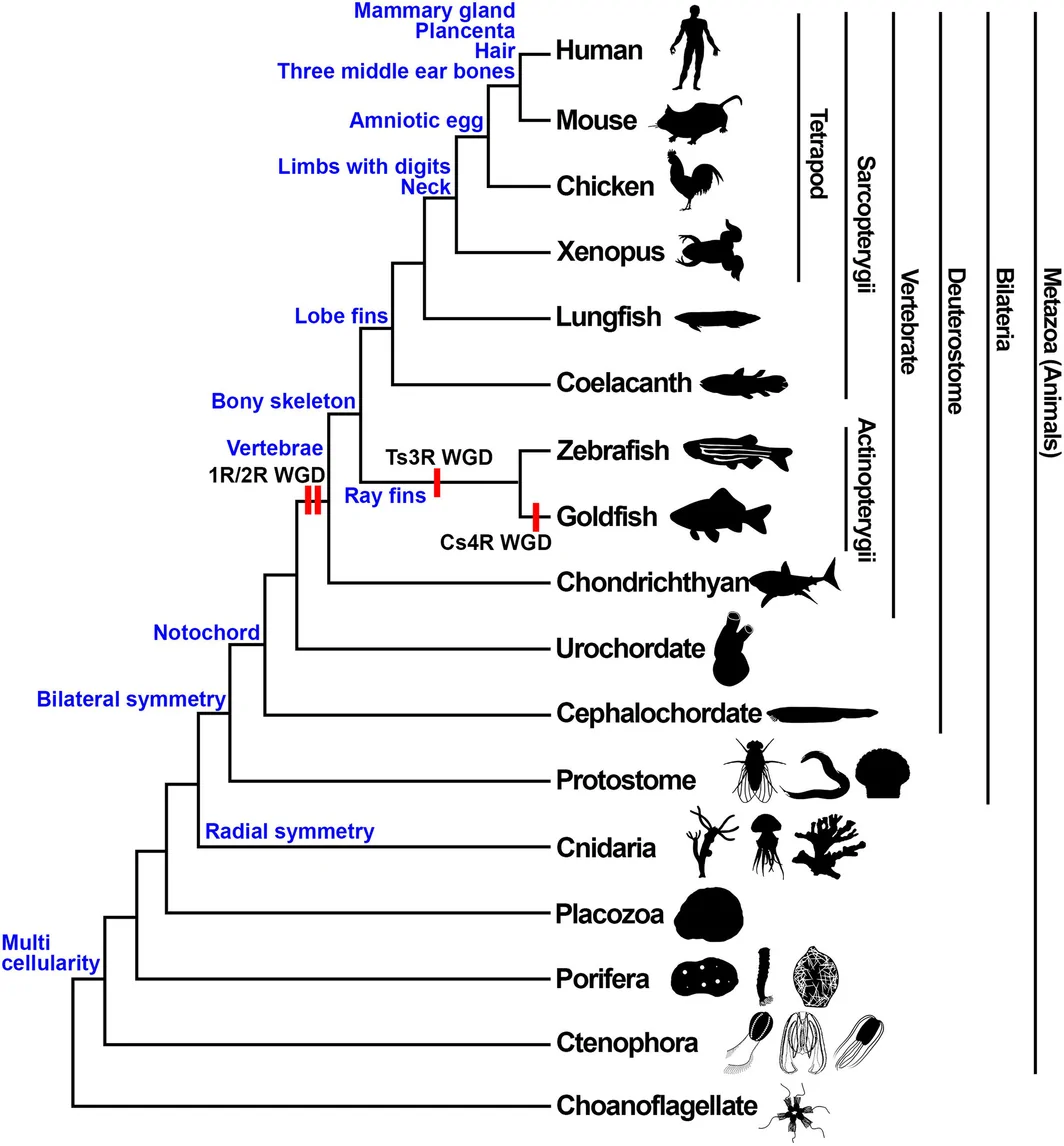
Phylogenetic relationships among major animal lineages This cladogram depicts the evolutionary relationships among key animal (Metazoa) groups. Red vertical bars on the respective branches indicate WGD events that contributed to vertebrate evolution: The two initial rounds at the base of vertebrates (1R/2R WGD), the teleost‐specific third round (Ts3R WGD), and the carp‐specific fourth round (Cs4R WGD) found in the goldfish lineage. Blue labels indicate key morphological and developmental traits that emerged along each lineage. The cladogram topology was constructed with reference to previous studies [52, 100, 101, 102, 103]. Animal silhouettes are from PhyloPic [58].

Teratology can now greatly benefit from advances in evolutionary and comparative genomics. The technological revolution that now makes chromosome‐level genome assembly widely achievable has advanced alongside genome editing techniques using CRISPR‐Cas9, together reshaping the landscape of developmental and evolutionary biology [91, 92, 93, 94]. This synergy is moving research beyond its traditional focus on classic model organisms [95, 96, 97, 98, 99]. While traditional models such as mice and zebrafish remain widely used, researchers are now studying other species with distinct traits or phylogenetic importance to address biological questions (Figure 2 and Table 1). Findings from one species can now be compared across others through comparative genomics using high‐quality genome assemblies. Comparisons between chromosome‐scale genome assemblies are revealing how genomes evolve at levels beyond individual genes or local regulatory elements [49, 50, 51, 52]. This framework offers new opportunities for analyzing the ultimate causes of congenital anomalies. In the following, we introduce three key research areas, each reflecting a different evolutionary timescale, that together offer new perspectives on teratology.

### 3.1 Vertebrate Appendage Patterning and Its Evolutionary Origins

Vertebrate limbs exhibit a conserved anatomical structure, with serial segments and distinct axes. Each limb is organized into a proximal stylopod (humerus or femur) that articulates with the girdle, an intermediate zeugopod (radius and ulna, or tibia and fibula), and a distal autopod with carpal/tarsal bones and typically five digits [104]. Limb development proceeds along three orthogonal axes including proximal–distal axis (from shoulder or hip to fingers or toes), anterior–posterior axis (from the thumb or radial side to the little finger or ulnar side), and dorsal–ventral axis (from the extensor side to the flexor side). These axes guide the orientation of bones and digits, and disruptions in their patterning manifest as congenital limb anomalies [105, 106]. For example, polydactyly is a common congenital anomaly characterized by supernumerary digits that can arise on the preaxial (radial/thumb) or postaxial (ulnar/little finger) borders [105, 106]. Syndactyly, in which adjacent digits are fused, represents a failure in digit separation and can range from soft‐tissue webbing to bony fusion. Phocomelia is a severe limb patterning defect, classically associated with thalidomide exposure during early pregnancy, that is characterized by severely underdeveloped or absent proximal limb segments, resulting in the hand or foot being positioned close to the trunk [107].

At the molecular level, vertebrate limb patterning is governed by highly conserved developmental pathways [108, 109, 110, 111]. During limb bud development, the apical ectodermal ridge (AER) at the dorsoventral ectodermal boundary directs patterning along the proximal–distal axis and governs limb outgrowth and differentiation, primarily via fibroblast growth factor (FGF) signaling [112]. The anterior–posterior axis, patterned by the posterior mesenchymal zone of polarizing activity (ZPA), depends on Sonic hedgehog (SHH) signaling [108, 109, 110, 111]. The dorsal‐ventral axis is mainly regulated by WNT signaling [108, 109, 110, 111]. Disruptions in these signaling pathways due to genetic mutations can lead to congenital limb anomalies, such as the formation of extra thumbs or great toes resulting from point mutations in the enhancer regulating Shh expression or from loss‐of‐function mutations in GLI3, a downstream transcription factor [105, 113].

**FIGURE 3:**
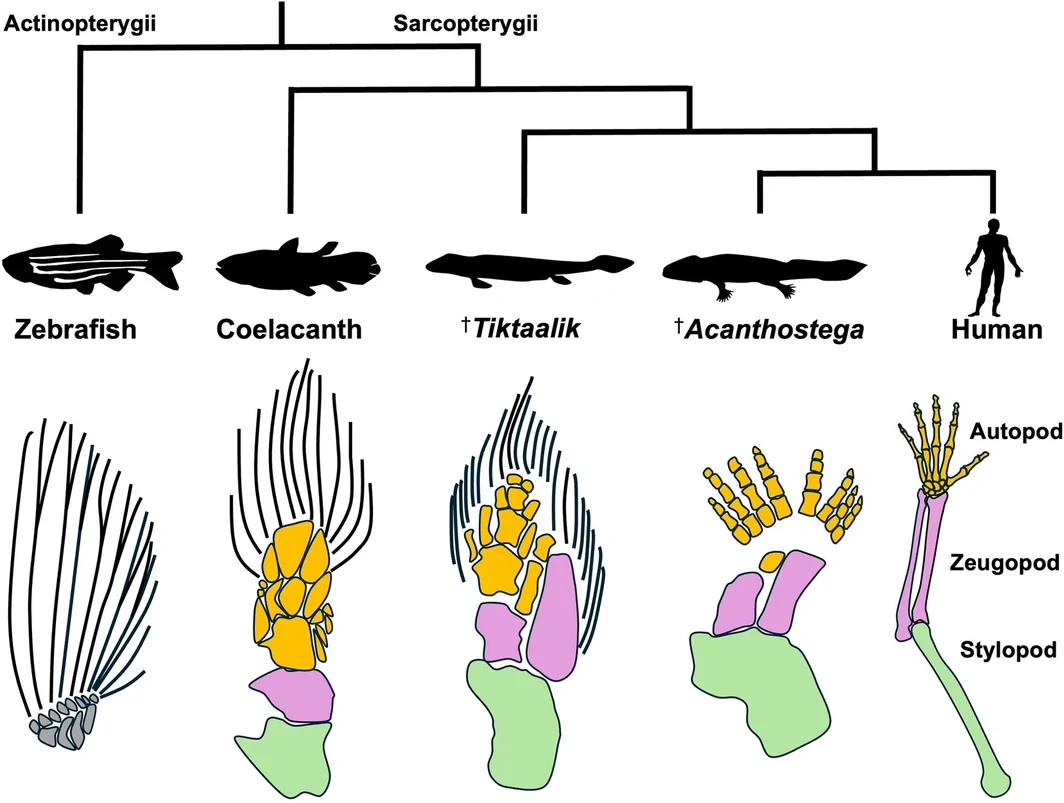
Evolutionary transition of paired appendages from fins to limbs in vertebrates. This figure depicts the paired appendage structures from zebrafish, coelacanth, extinct transitional species (†*Tiktaalik* and †*Acanthostega*), and human. Homologous skeletal elements are color‐coded consistently across species. Illustrations were drawn by the author based on previous studies [113, 117, 118, 119, 120]. Animal silhouettes are from PhyloPic [58].

Paired appendages, a distinctive feature of vertebrates, are absent in extant invertebrate chordates (Figure 2), such as amphioxus (cephalochordate) and sea squirts (urochordates) [114]. Important early fossil evidence comes from Paleozoic jawless vertebrates, such as *Tujiaaspis vividus* from the Silurian period (444–419 million years ago [MYA]) [115], which developed paired fin‐like lateral structures, and *Kolymaspis sibirica* from the early Devonian period (419–359 MYA), which provides fossil evidence relevant to the origin of the vertebrate pectoral girdle [116]. Later, in the late Devonian, lobe‐finned fishes (early sarcopterygians, Figure 3) exhibited limb structures that bridge the “fish‐to‐tetrapod transition.” The fossil *Eusthenopteron foordi* and the famous transitional form *Tiktaalik roseae* possessed the stylopod and zeugopod of tetrapods [113, 121]. Not long after, some of the earliest tetrapods appeared, such as *Ichthyostega stensioei* and *Acanthostega gunnari*, both of which exhibited more than five digits [113, 117, 121, 122, 123] (Figure 3). The standard five‐digit limb of extant tetrapod lineages (the pentadactyl limb) [118] thus arose later as digit number stabilized to five in many tetrapod lineages. Although most animals exhibiting such transitional limb features have gone extinct, lungfish and coelacanths have survived as basally branching sarcopterygians (Figure 2). Their genomes provide important molecular evolutionary insights into ancestral vertebrate limb developmental programs [123, 124, 125, 126, 127] and may help explain why congenital limb anomalies recur in a limited range of patterns shaped by developmental constraints. Comparative analysis of conserved noncoding elements across vertebrate genomes may further improve the identification and interpretation of regulatory or structural variants underlying unexplained limb anomalies.

### 3.2 Whole‐Genome Duplications (WGDs) as a Source of Evolutionary Novelty

Major evolutionary innovations are often associated with increases in genomic complexity [128, 129, 130]. One of the most profound mechanisms for generating such genome complexity is WGD, an event where an entire chromosome set is doubled [131, 132]. In 1970, Susumu Ohno proposed that the evolutionary success and anatomical complexity of vertebrates were facilitated by two ancient rounds of WGD (2R hypothesis) early in their history [128]. Advances in genomics have confirmed this hypothesis, showing that the genomes of jawed vertebrates, including humans, still have the signature of ancient WGDs, with duplicated chromosomal segments reflecting their paleotetraploid ancestry [50]. However, WGD can be viewed as a significant evolutionary trade‐off. While WGD could provide a wealth of raw genetic material for evolutionary innovation [128], it also poses immediate challenges to genomic stability and the precise regulation of gene dosage, potentially creating new vulnerabilities in developmental systems [133].

The 1R/2R WGDs occurred at the base of the vertebrate lineage more than 500 million years ago, and much of the subsequent gene losses and genome rearrangements have obscured the initial impact [134, 135, 136]. Teleosts also underwent a lineage‐specific third round (3R) of WGD approximately 300 MYA or before [136, 137], which provides a clearer view of the effects of WGD [138, 139, 140]. The duplicated genes, or ohnologs, generated by WGDs can follow several evolutionary fates [128, 129, 141]. Many ohnologs undergo nonfunctionalization due to deleterious mutations and are eventually lost. Others are retained through subfunctionalization, where ancestral functions are partitioned, or neofunctionalization, where one copy acquires a novel function. Additionally, dosage selection can preserve both copies when maintaining gene balance is advantageous for cellular function.

WGDs and subsequent evolutionary diversification of ohnologs facilitate the evolution of more complex and specialized developmental networks. An example of such diversification is seen in the vertebrate Otx family of homeobox transcription factors. In vertebrates, the ancient ohnologs Otx2 and Crx are both essential for proper retinal development [142, 143, 144, 145, 146] but are not functionally interchangeable [147]. *Otx2* is essential for photoreceptor cell fate determination [142] and plays a distinct role in the maturation of photoreceptor and bipolar cells [148]. In contrast, Crx is specifically expressed in photoreceptor cells, and its deletion impairs photoreceptor maturation, leading to photoreceptor degeneration [143, 144, 145, 146]. This functional specialization after the vertebrate 1R/2R WGD contributes to the complexity of the retinal architecture. The trade‐off is that this increased complexity also creates more potential points of failure. Indeed, mutations in human OTX2 and CRX result in different genetic disorders [144, 149].

**FIGURE 4:**
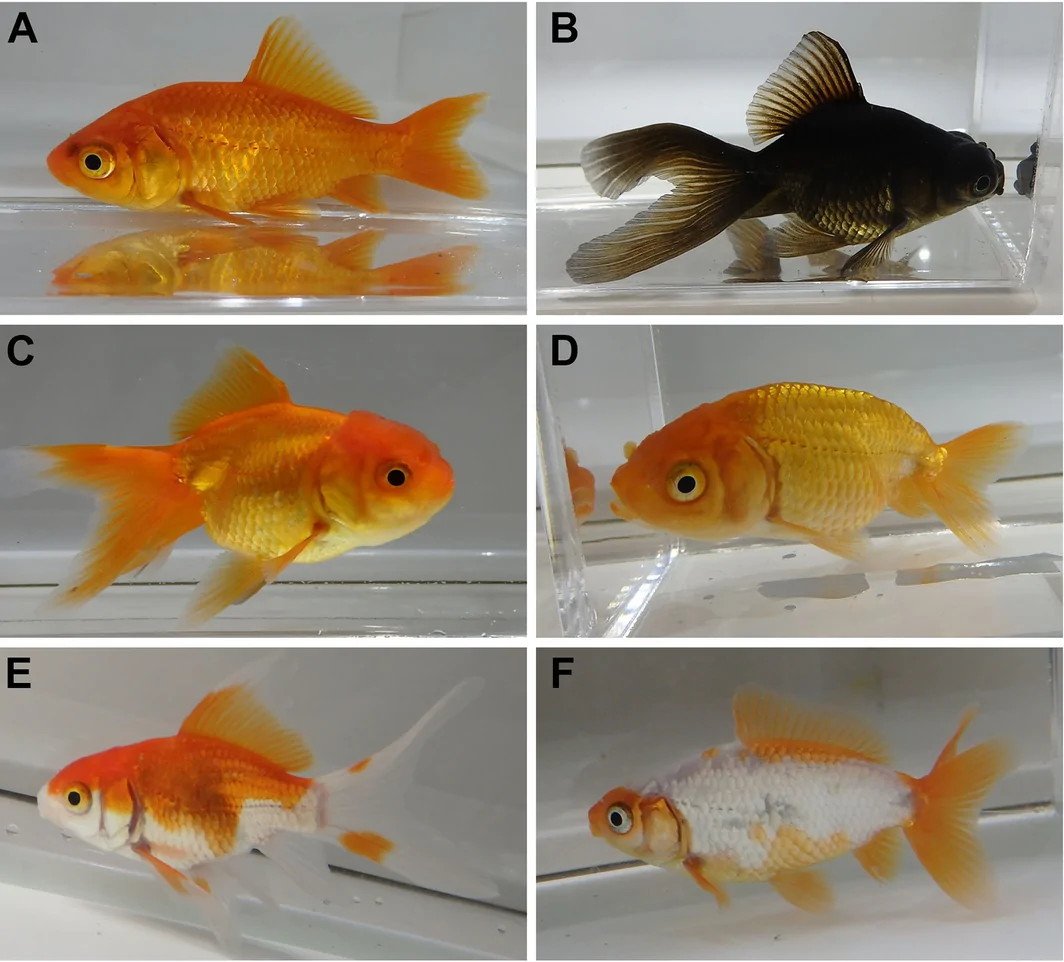
Morphological diversity of the ornamental goldfish strains. Representative strains of ornamental goldfish exhibit the extensive phenotypic diversity, such as eye size, cranial morphology, body shape, and coloration [33, 96]. Each figure (A–F) shows Wakin (A), Black Telescope‐eye (B), Oranda (C), Ranchu (D), Tamasaba (E), and Jikin (F). Photographs were generously provided by Yoshihiro Omori of Hiroshima University.

To investigate the early consequences of WGD, an ideal model is an organism that has undergone a more recent WGD. The domesticated goldfish ( *Carassius auratus* ) provides such a model. Goldfish exhibit remarkable phenotypic diversity (Figure 4), and underwent an allotetraploidization event (interspecific hybridization followed by WGD) approximately 14 MYA (Cs4R, carp‐specific WGD) [100]. The goldfish subgenomes, each consisting of a set of chromosomes derived from one of the two parental species involved in allotetraploidization, exhibit distinct patterns of genome evolution, called asymmetric subgenome evolution [33, 150]. One subgenome remains more similar to the ancestral state (dominant subgenome), whereas the other shows more gene losses and changes in levels of gene expression (nondominant subgenome) [33, 150]. Of note, a genome‐wide association study on various goldfish phenotypes suggested that genetic variants associated with phenotypes tend to be more prevalent in the nondominant subgenome [33]. This suggests that the accumulation of mutations in the asymmetrically evolved subgenomes underlies the phenotypic diversity in goldfish strains. Given that the 2R‐WGD in jawed vertebrates is also thought to have resulted from an allotetraploidization event [50, 151], the asymmetric subgenome evolution and phenotypic diversity observed in goldfish may offer insights into the diversification of body plans in vertebrates, including humans. More broadly, the increased complexity of developmental gene networks following WGD may have promoted evolutionary innovation while also increasing vulnerability to gene‐dosage imbalance and disruption of specialized ohnolog functions. However, this evolutionary perspective should not be taken to imply that WGD is equivalent to pathogenic copy‐number variants in individual patients. WGD involves duplication of the entire genome followed by long‐term gene loss, functional divergence, and network reorganization, whereas pathogenic copy‐number variants generally involve localized deletions or duplications that directly alter gene dosage within an individual genome. Rather, species with more recent WGD events, such as goldfish, may help identify duplicated genes and developmental networks that are particularly sensitive to dosage imbalance.

### 3.3 Deep Homology and the Anciently Active Transposable Elements of Animal Chromosomes

The phylum Cnidaria, which includes organisms such as sea anemones, corals, and hydra, occupies a key phylogenetic position. It is the sister group to the bilateria, a clade that comprises the vast majority of animals, including vertebrates [99, 152]. Although cnidarians have a relatively simple body plan, usually consisting of a radially symmetrical polyp with two germ layers, they possess a surprisingly complex gene toolkit that closely resembles that of bilaterians [153, 154, 155]. This makes cnidarians particularly valuable for reconstructing the genomic and developmental features of the last common ancestor of all animals [154, 156].

Cnidarians also exhibit remarkable biological traits. For instance, Hydra is a freshwater cnidarian with the earliest record of animal regeneration described by Trembley [157]. This animal shows extreme regenerative abilities [152, 158] and even exhibits biological immortality [159, 160]. These features offer unique opportunities to investigate fundamental principles of stem cells, senescence, and pattern formation, all of which are relevant to the study of congenital anomalies, regenerative medicine, and geriatric medicine.

**FIGURE 5:**
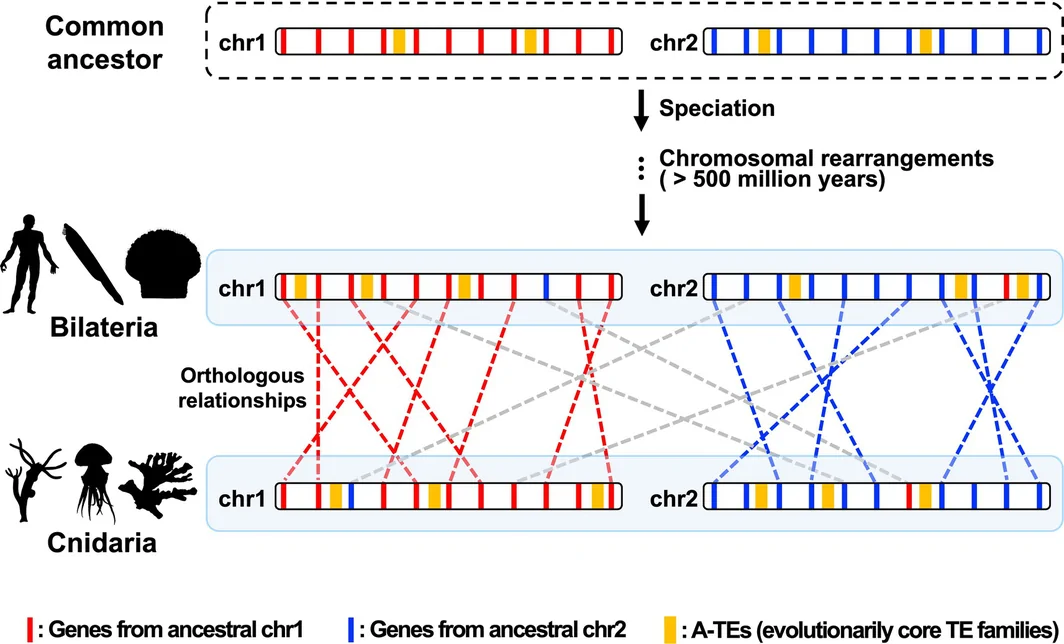
Chromosomal homology and evolutionarily conserved core TE families in animal genomes Homologous gene sets have remained on the same chromosomes across evolutionarily distant animals, forming ancestral linkage groups (represented by red and blue gene sets) [51]. In addition, A‐TEs comprise an evolutionarily conserved TE core, indicated by orange bars, which appears to have remained active both in Bilateria and Cnidaria [49]. Animal silhouettes are from PhyloPic [58].

Although early genome sequencing projects revealed that animal gene repertoires are widely conserved across major clades [101, 102, 153, 155, 161], it remained unclear whether any general principles underlie genome organization at the chromosome scale. However, this situation has changed in recent years, as chromosome‐level genome assemblies have become available for a broader range of animal lineages. Comparisons of the chromosome‐level genome assemblies have revealed chromosome‐scale conservation of synteny among bilaterians, cnidarians, and sponges [51, 52] (Figure 5). This means that sets of many homologous genes remained on the same chromosomes across evolutionarily distant animals (ancestral linkage groups, ALGs), even if their exact order on chromosomes differed [51]. ALGs are considered to reflect the chromosomal components of the last common ancestor of all animals and have remained largely conserved over more than half a billion years of animal evolution. The long‐term conservation of gene linkages may suggest functional relevance, potentially maintained by purifying selection. The arrangement of genes on chromosomes can influence their three‐dimensional positioning within the nucleus and may facilitate coordinated regulation of genes involved in related biological processes [162]. From this viewpoint, it is possible that certain congenital anomalies could be linked not only to mutations in individual genes but also to disruptions in the large‐scale genome organization.

Another important insight from cnidarian genomics concerns transposable elements (TEs), often referred to as “jumping genes” [163]. The genome of *Hydra vulgaris* is particularly rich in TEs and exhibits substantial genome expansion [153, 164]. Recently, telomere‐to‐telomere genome assemblies of two hydra strains have been generated, enabling high‐resolution analyses of TE genomic dynamics [49]. These analyses revealed that sets of TE families (active TEs, A‐TEs) are selectively active and tend to insert into specific genomic regions depending on the stem cell lineages. Notably, A‐TEs comprise an evolutionarily conserved TE core, which appears to have remained active across many animal phyla, not only in Hydra [49]. This suggests that these elements were already present in the earliest animals and have been maintained throughout evolution. In this context, some TEs might serve beneficial functions, such as stabilizing chromosome structure or regulating nearby genes. The deep conservation of chromosomal linkages and A‐TE families raises the possibility that their disruption or dysregulation could perturb developmental gene regulation and increase susceptibility to structural anomalies. Combining cross‐species maps of conserved genome organization with long‐read sequencing of human populations may help assess the pathogenic significance of large structural variants that remain difficult to interpret clinically.

## 4 Future Directions: Toward a “Next‐Generation Teratology”

Next‐generation teratology, grounded in evolutionary and comparative genomics, offers a broadened approach to studying congenital anomalies (Figure 1). Rather than focusing solely on proximate causes, such as *how* genetic mutations or environmental exposures lead to congenital anomalies, this perspective also asks *why* developmental systems are so susceptible to disruption, resulting in congenital anomalies. By incorporating concepts such as evolutionary trade‐offs, environmental mismatch, and developmental constraints, teratology gains a framework that complements traditional clinical and experimental insights. Importantly, this perspective is no longer merely conceptual. It is now empirically grounded through recent advances in chromosome‐level genome assembly and large‐scale comparative analyses. These technological developments allow the structure and evolution of developmental gene networks to be investigated at unprecedented resolution through comparative analysis of genomic data from humans and diverse animal species (Figure 1) [165, 166].

A major objective of next‐generation teratology will be to map the conserved genomic architecture underlying vertebrate development, whose disruption contributes to human congenital anomalies (Figure 1). Large‐scale genome projects enable comparisons across species at the chromosome level. These comparisons can reveal conserved noncoding elements, syntenic blocks, and topologically associating domains [167, 168, 169, 170] that define the structural core of developmental regulation. In parallel, chromosome‐level assemblies of individual human genomes are becoming increasingly available, enabling the detection of large structural variants within the human population that intersect with these conserved regulatory regions. Identifying variants linked to congenital anomalies in such evolutionarily stable regions can strengthen causal inferences, even prior to functional validation.

Concepts from evolutionary biology can help to understand *why* certain congenital anomalies emerge. For instance, vertebrates underwent two rounds of WGDs, and some ohnologs are retained in vertebrate genomes. While some of these ohnologs may have facilitated the evolution of novel traits by increasing the complexity of developmental gene networks, this increased complexity can also make the system more fragile, raising the risk of developmental defects as an evolutionary trade‐off [171, 172]. The principle of antagonistic pleiotropy is evident in cases where genes are positively selected for beneficial functions, such as neocortex expansion in mammals. These same genes, however, may also carry variants that increase susceptibility to congenital anomalies, suggesting how an adaptation that is advantageous in one context can simultaneously introduce a vulnerability in another. The concept of environmental mismatch further contributes to understanding developmental vulnerability. Genetic variants that were presumably adaptive in ancestral environments may interact negatively with novel modern exposures, such as synthetic chemicals or contemporary diets. Experimentally testing these gene–environment interactions may explain why certain congenital anomalies are observed under present‐day conditions. Analyses of ancient human DNA and reconstructions of past environments may also provide useful clues [173, 174].

Artificial intelligence may also contribute to next‐generation teratology through the prediction of drug teratogenicity. For example, embryoTox, a graph‐based model using small‐molecule structural signatures, showed high performance in predicting drug teratogenicity. The reported area under the receiver operating characteristic curve (AUC), a measure of classification performance, was 0.96 in internal cross‐validation and 0.82 in an independent blind‐test dataset [175]. FATE‐MAP, which combines human gastruloid‐based phenotypic mapping with deep learning and mechanistic modeling, achieved an overall accuracy of 72.7% and a balanced accuracy of 74.8% in a 33‐drug benchmark panel [176]. These studies suggest that AI‐based approaches can help prioritize compounds with potential teratogenic risk. However, such models should be regarded as screening or prioritization tools rather than standalone clinical tests, and their future use will require larger and more representative datasets, independent external validation, interpretable outputs, and careful evaluation of false‐negative predictions [177].

As teratology moves into this new phase, ethical considerations will become increasingly important. The ability to predict congenital anomaly risk from genome data raises complex issues around prenatal testing, reproductive decision‐making, and perceptions of disability [178]. The growing detection of genetic variants of uncertain significance in conserved genomic regions may also complicate genetic counseling. Furthermore, advances in genome editing raise questions about the boundary between therapy and enhancement, informed consent, and fair access [179, 180, 181]. The use of large international genomic datasets in teratology underscores the need for robust governance around privacy, data sharing, and incidental findings [182, 183].

Despite these challenges, next‐generation teratology holds great promise. With the aid of evolutionary and comparative genomics, researchers can extend the study of congenital anomalies beyond proximate mechanisms to uncover why certain patterns of disruption recur across species and contexts. This integrated perspective may ultimately lead to improved strategies for diagnosing, managing, and preventing congenital anomalies, while deepening our understanding of developmental vulnerability.

## Funding

This work was supported by a JSPS Overseas Research Fellowship from the Japan Society for the Promotion of Science and a fellowship from the Takeda Science Foundation.

## Ethics Statement

This article is a review article and does not involve any original experiments, human participants, animal subjects, clinical data collection, or analyses requiring ethics approval. Therefore, ethical approval was not required. The manuscript was prepared with careful consideration of ethical issues relevant to the discussion of congenital anomalies, developmental disorders, and genomic information.

## Conflicts of Interest

The author declares no conflicts of interest.

## Supporting information


**Figure S1:** Pathological‐anatomical collection in the *Narrenturm*.
**Figure S2:** Workflow for chromosome‐level genome assembly.

## Data Availability

Data sharing not applicable to this article as no datasets were generated or analyzed during the current study.
