# Supplementary material for: Toward Next‐Generation Teratology: Understanding Developmental Vulnerability Through Evolutionary and Comparative Genomics
Source: Congenit Anom (Kyoto). 2026 Aug 26;66(1):e70074. doi: 10.1002/cga.70074 (PMC13507876; doi:10.1002/cga.70074)
Supplement: Supplementary file 1 [file CGA-66-0-s001.docx]

**Supplementary figures**

**Fig. S1. Pathological-anatomical collection in the *Narrenturm***

(A) The *Narrenturm* in Vienna, exhibiting one of the largest teratological collections in Europe. The origin of the pathological-anatomical collection in Vienna can be traced back to the foundation of pathological-anatomical museum at the Institute of Pathology in 1796^1^. Between 1834 and 1874, Carl von Rokitansky undertook large-scale curation and expansion of the collection, increasing it to 4,833 specimens and compiling a new catalogue^1^. This effort also formed the foundation of his seminal works in pathological anatomy^1–4^. In 1971, the pathological-anatomical collection was relocated to the *Narrenturm*, originally a psychiatric hospital. Today, the collection in the *Narrenturm* comprises more than 50,000 specimens, including 1,200 teratological specimens, making it one of the largest teratological collections in Europe^1^. Photo taken by the author in July 2025. (B) Bust of Carl von Rokitansky, a pioneer of pathological anatomy who significantly contributed to the development of the pathological-anatomical collection. The marble bust is located in the *Arkadenhof* of the University of Vienna. Image reproduced without modification from Wikimedia Commons (file name: Carl von Rokitansky (Nr. 54) Bust in the Arkadenhof, University of Vienna-9281.jpg).

**Fig. S2. Workflow for chromosome-level genome assembly**

This figure illustrates the general workflow for achieving chromosome-level genome assembly. Within the nucleus, the genome is spatially organized into distinct chromosome territories, and even within each territory, chromosomal regions are arranged in a non-random, spatially constrained manner reflecting their functional and structural interactions. Third-generation sequencing technologies produce long reads that span repetitive regions, allowing for highly contiguous assemblies. In de novo assembly, overlaps between long reads are detected to generate contiguous sequences (contigs) without the need for a reference genome. Meanwhile, the Hi-C sequencing captures the three-dimensional chromatin interactions. Hi-C scaffolding uses contact frequency data to estimate the relative positions and orientations of contigs along chromosomes, and then orders and joins them into scaffolds, thereby reconstructing the genome sequence at the chromosomal scale.

**Supplementary Note**

**Rapid advances in genome sequencing technology**

The integration of an evolutionary framework into teratology is not merely a conceptual shift but is now enabled by genome sequencing technologies. Over the past half-century, these technologies have transformed both evolutionary biology and clinical genetics into increasingly quantitative, experimental, and data-driven sciences, while expanding analyses from individual genes to entire genomes^5–8^. A brief historical overview is therefore useful because current clinical and comparative genomic approaches emerged through a stepwise expansion in the scale and types of genomic variation that could be detected and interpreted. Consequently, congenital anomalies can now be investigated not only at the level of individual genes but also through complex structural variants, noncoding regulatory changes, and evolutionarily conserved genomic architectures.

***From Sanger sequencing to the Human Genome Project***

The origins of evolutionary biology can be traced to the macroscopic observations made by early naturalists. These begin with the taxonomic systems of Aristotle and Carl von Linné^9^. The field further developed through the voyages of Charles Darwin, whose encounters with diverse species, particularly in the Galápagos Islands, inspired the theory of evolution by natural selection^10,11^.

In the first half of the 20th century, biology underwent a major shift through the rise of biochemistry, which enabled molecular-level comparisons between species using enzyme activity and immunological techniques^12^. Subsequently, following the development of protein sequencing technology in the 1950s, this molecular data were combined with population genetics in the 1960s, giving rise to the new field of molecular evolution^13,14^.

The discovery of the DNA double helix in 1953 provided a foundation for further advances in biology^15^. In 1977, Frederick Sanger developed the chain-termination method of DNA sequencing^16^, which allowed direct, base-by-base comparison of genes between species. The Human Genome Project (HGP) started in 1990 with a massive international effort. This initiative led to the development of automated capillary sequencers^17^ and advanced computational tools, known as genome assemblers including Celera Assembler^18^, which were used to piece together the sequenced fragments. The human genome sequence by the HGP was first announced as a draft in 2001^19^ and later completed in 2004^20^. A few years earlier or around the same time, the genomes of key model organisms such as yeast (*Saccharomyces cerevisiae*)^21^, fruit fly (*Drosophila melanogaster*)^22^, and nematode (*Caenorhabditis elegans*)^23^ were also sequenced by Sanger sequencing.

Through genome sequencing, biology shifted from a focus on individual genes to a broader view of biological systems, leading to the emergence of omics approaches aimed at understanding how large numbers of molecular components such as genes and proteins work together in complex networks^24–26^. However, these early genomes, assembled from Sanger sequencing reads, were still mosaics of fragmented sequences. These were first assembled into “contigs” and then linked into “scaffolds”, but often contained numerous gaps and failed to represent whole chromosomal sequences.

***The next-generation sequencing revolution***

Around 2005, a new wave of next-generation sequencing (NGS) technologies, primarily based on sequencing-by-synthesis (e.g., 454 pyrosequencing and Solexa/Illumina platforms), revolutionized genomics by enabling massively parallel sequencing^27–29^. This led to a precipitous drop in sequencing costs, famously outpacing Moore's Law, and an exponential increase in data output^27,28^.

The massive throughput of NGS technologies is particularly powerful for a method known as genome resequencing^27,30^. In this method, sequencing reads are aligned to a reference genome. By comparing the nucleotide sequences of the reads with those of the reference, genetic markers such as single-nucleotide polymorphisms (SNPs) can be identified on a large scale^27,30^. Coupled with the dramatic reduction in sequencing costs, this approach has facilitated population-scale investigations of genomic diversity. It has been applied not only to humans but also to a wide range of non-human animals and continues to be used to analyze population genetic structure^31–35^ and identify genetic loci associated with diverse traits in species^36^.

However, a fundamental challenge in genome assembly persisted. Short-read sequencing technologies, typically producing reads of 35–300 base pairs, made the de novo assembly of large, repetitive eukaryotic genomes extremely difficult^37^. A notable example was the de novo assembly of the giant panda genome, which was one of the first large mammalian genomes assembled exclusively using short-read Illumina data^38^. This was achieved with the SOAPdenovo assembler, which utilizes a de Bruijn graph algorithm^39^ and required extensive paired-end libraries with varying insert sizes to span gaps. Although this study demonstrated that a large mammalian genome could be assembled using short-read sequencing alone, the resulting draft genome had a contig N50 of 40 kilobases and an N50 scaffold size of 1.3 megabases^38^, illustrating the limitations of short-read NGS technologies in resolving complex and repetitive genomic structures.

***The era of chromosome-level genome assemblies***

The development of long-read sequencing and high-throughput chromosome conformation capture (Hi-C) sequencing enabled chromosome-level genome assemblies to be routinely achieved in the late 2010s (Fig. S2). Third-generation sequencing technologies, developed by Pacific Biosciences and Oxford Nanopore Technologies, enable real-time sequencing of single DNA molecules^29^. Unlike first-generation Sanger sequencing and second-generation short-read NGS, these platforms produce exceptionally long reads, ranging from tens of thousands to even millions of base pairs. These long reads can span repetitive elements that typically break short-read assemblies, enabling the generation of contigs on the megabase scale. In some cases, these contigs can approach tens of megabases in length, which is comparable to the size of chromosome arms in typical animal genomes^40^. The Hi-C technology captures the three-dimensional proximity of DNA segments within the nucleus^41^. By analyzing the contact frequencies between contigs, Hi-C data allow them to be ordered and oriented into chromosome-length scaffolds, a process known as Hi-C scaffolding^42,43^ (Fig. S2).

The combination of long-read sequencing for contiguity and Hi-C for scaffolding has made it possible to routinely generate high-quality, chromosome-level genome assemblies, even at the level of individual laboratories. Several large international initiatives, including the Earth BioGenome Project (EBP)^44^, the Darwin Tree of Life Project (DToL)^45^, and the Vertebrate Genomes Project (VGP)^46^ aim to produce high-quality, chromosome-level reference genomes using standardized methods (Fig. 1). EBP and DToL target all extant eukaryotic species, while VGP focuses specifically on all vertebrate species. These efforts are helping to establish a broad comparative framework for studying the evolution of life on Earth, and enabling the identification of conserved developmental programs from a genomic perspective.

Chromosome-level assembly technologies are also facilitating population genome assembly approaches in humans that reconstruct individual genomes without reference genome guidance^42,47^ (Fig. 1). By comparing chromosome-scale assemblies from multiple individuals, this strategy can detect large structural variants including insertions, deletions, inversions, and rearrangements that are often missed in traditional genome resequencing. These variants may contribute to congenital anomalies for which no causative mutations have been identified, providing new opportunities to uncover hidden pathogenic genomic changes.

**Supplementary references**

1.	Winter, E., Feigl, W. & Wiltschke-Schrotta, K. *Picturing Diseases: The pathological-anatomical collection at the Narrenturm*. (Naturhistorisches Museum Wien, 2021).

2.	Boer, L. L. *et al.* History and highlights of the teratological collection in the Narrenturm , Vienna (Austria). *Am. J. Med. Genet. Part A* **191**, 1301–1324 (2023).

3.	Ortiz-Hidalgo, C. Carl von Rokitansky, the Linné of pathological anatomy. *Gac. Med. Mex.* **156**, (2023).

4.	Winter, E., Teschler-Nicola, M., Macfelda, K. & Vohland, K. The pathological anatomical collection of the Natural History Museum Vienna. *Wiener Medizinische Wochenschrift* **174**, 265–278 (2024).

5.	Brookfield, J. F. Y. Evolution Is a Quantitative Science. *PLoS Biol.* **8**, e1000381 (2010).

6.	Song, L. & Wangs, W. Genomes and evolutionary genomics of animals. *Curr. Zool.* **59**, 87–98 (2013).

7.	Kondrashov, A. S. Comparative genomics and evolutionary biology. *Curr. Opin. Genet. Dev.* **9**, 624–629 (1999).

8.	Medina, M. Genomes, phylogeny, and evolutionary systems biology. *Proc. Natl. Acad. Sci.* **102**, 6630–6635 (2005).

9.	Yucesan, E. Carl von Linné: The Development of the Idea of Binomial Nomenclature. *J. Med. Biogr.* **32**, 51–56 (2024).

10.	Darwin, C. *On the Origin of Species*. (John Murray., 1859).

11.	Ayala, F. J. Darwin and the scientific method. *Proc. Natl. Acad. Sci.* **106**, 10033–10039 (2009).

12.	K., H. D. An Introduction to Comparative Biochemistry. *Nature* **139**, 819–819 (1937).

13.	Lee, M. S. Y. & Ho, S. Y. W. Molecular clocks. *Curr. Biol.* **26**, R399–R402 (2016).

14.	KIMURA, M. Evolutionary Rate at the Molecular Level. *Nature* **217**, 624–626 (1968).

15.	WATSON, J. D. & CRICK, F. H. C. Molecular Structure of Nucleic Acids: A Structure for Deoxyribose Nucleic Acid. *Nature* **171**, 737–738 (1953).

16.	Sanger, F. *et al.* Nucleotide sequence of bacteriophage φX174 DNA. *Nature* **265**, 687–695 (1977).

17.	Zhang, J. *et al.* Use of Non-Cross-Linked Polyacrylamide for Four-Color DNA Sequencing by Capillary Electrophoresis Separation of Fragments up to 640 Bases in Length in Two Hours. *Anal. Chem.* **67**, 4589–4593 (1995).

18.	Myers, E. W. *et al.* A Whole-Genome Assembly of Drosophila. *Science (80-. ).* **287**, 2196–2204 (2000).

19.	Lander, E. S. *et al.* Initial sequencing and analysis of the human genome. *Nature* **409**, 860–921 (2001).

20.	Finishing the euchromatic sequence of the human genome. *Nature* **431**, 931–945 (2004).

21.	Goffeau, A. *et al.* Life with 6000 Genes. *Science (80-. ).* **274**, 546–567 (1996).

22.	Adams, M. D. *et al.* The Genome Sequence of Drosophila melanogaster. *Science (80-. ).* **287**, 2185–2195 (2000).

23.	C. elegans Sequencing Consortium. Genome Sequence of the Nematode C. elegans : A Platform for Investigating Biology. *Science (80-. ).* **282**, 2012–2018 (1998).

24.	Lander, E. S. Initial impact of the sequencing of the human genome. *Nature* **470**, 187–197 (2011).

25.	Kitano, H. Systems Biology: A Brief Overview. *Science (80-. ).* **295**, 1662–1664 (2002).

26.	Joyce, A. R. & Palsson, B. Ø. The model organism as a system: integrating ‘omics’ data sets. *Nat. Rev. Mol. Cell Biol.* **7**, 198–210 (2006).

27.	Shendure, J. *et al.* DNA sequencing at 40: past, present and future. *Nature* **550**, 345–353 (2017).

28.	Koboldt, D. C., Steinberg, K. M., Larson, D. E., Wilson, R. K. & Mardis, E. R. The Next-Generation Sequencing Revolution and Its Impact on Genomics. *Cell* **155**, 27–38 (2013).

29.	Goodwin, S., McPherson, J. D. & McCombie, W. R. Coming of age: ten years of next-generation sequencing technologies. *Nat. Rev. Genet.* **17**, 333–351 (2016).

30.	Davey, J. W. *et al.* Genome-wide genetic marker discovery and genotyping using next-generation sequencing. *Nat. Rev. Genet.* **12**, 499–510 (2011).

31.	Freedman, A. H. *et al.* Genome Sequencing Highlights the Dynamic Early History of Dogs. *PLoS Genet.* **10**, e1004016 (2014).

32.	Kon, T. *et al.* Whole-genome resequencing of large yellow croaker (Larimichthys crocea) reveals the population structure and signatures of environmental adaptation. *Sci. Rep.* **11**, 11235 (2021).

33.	Li, X. *et al.* Whole-genome resequencing of wild and domestic sheep identifies genes associated with morphological and agronomic traits. *Nat. Commun.* **11**, 2815 (2020).

34.	Jiang, L. *et al.* Whole-genome sequencing of endangered Zhoushan cattle suggests its origin and the association of MC1R with black coat colour. *Sci. Rep.* **11**, 17359 (2021).

35.	Pool, J. E. *et al.* Population Genomics of Sub-Saharan Drosophila melanogaster: African Diversity and Non-African Admixture. *PLoS Genet.* **8**, e1003080 (2012).

36.	Kon, T. *et al.* The Genetic Basis of Morphological Diversity in Domesticated Goldfish. *Curr. Biol.* **30**, 2260-2274.e6 (2020).

37.	Alkan, C., Sajjadian, S. & Eichler, E. E. Limitations of next-generation genome sequence assembly. *Nat. Methods* **8**, 61–65 (2011).

38.	Li, R. *et al.* The sequence and de novo assembly of the giant panda genome. *Nature* **463**, 311–317 (2010).

39.	Compeau, P. E. C., Pevzner, P. A. & Tesler, G. How to apply de Bruijn graphs to genome assembly. *Nat. Biotechnol.* **29**, 987–991 (2011).

40.	Kon-Nanjo, K. *et al.* Chromosome-level genome assembly of Hydractinia symbiolongicarpus. *G3 (Bethesda).* **13**, (2023).

41.	Bonev, B. & Cavalli, G. Organization and function of the 3D genome. *Nat. Rev. Genet.* **17**, 661–678 (2016).

42.	Li, H. & Durbin, R. Genome assembly in the telomere-to-telomere era. *Nat. Rev. Genet.* **25**, 658–670 (2024).

43.	Garg, S. Towards routine chromosome-scale haplotype-resolved reconstruction in cancer genomics. *Nat. Commun.* **14**, 1358 (2023).

44.	Lewin, H. A. *et al.* Earth BioGenome Project: Sequencing life for the future of life. *Proc. Natl. Acad. Sci.* **115**, 4325–4333 (2018).

45.	Blaxter, M. *et al.* Sequence locally, think globally: The Darwin Tree of Life Project. *Proc. Natl. Acad. Sci.* **119**, (2022).

46.	Rhie, A. *et al.* Towards complete and error-free genome assemblies of all vertebrate species. *Nature* **592**, 737–746 (2021).

47.	Gao, Y. *et al.* A pangenome reference of 36 Chinese populations. *Nature* **619**, 112–121 (2023).
